# Social ties influence teamwork when managing clinical emergencies

**DOI:** 10.1186/s12909-020-1953-8

**Published:** 2020-03-04

**Authors:** Maria B. Rasmussen, Martin G. Tolsgaard, Peter Dieckmann, Doris Østergaard, Jonathan White, Pernille Plenge, Charlotte V. Ringsted

**Affiliations:** 1 Department of Obstetrics and Gynecology, University hospital Sealand, Roskilde Hospital. Sygehusvej 10, 4000 Roskilde, Denmark; 2 Copenhagen Academy for Medical Education and Simulation (CAMES), Capital Region of Denmark Centre for Human Resource, Blegdamsvej 9, 2100 København Ø, Copenhagen, Denmark; 3 Intensive Care Unit 4131, University Hospital Rigshospitalet, Blegdamsvej 9. 2100, Købenahvn Ø, Denmark; 4 Faculty of Health, Aarhus University, Ndr. Ringgade 1, 8000 Aarhus, Denmark

**Keywords:** Social ties, Team performance., Team communication., Team coordination., Management of medical emergencies.

## Abstract

**Background:**

Our current understanding of medical team competence is traditionally influenced by an individualistic perspective focusing on individual team members’ knowledge, skills as well as on effective communication within the team. However, team dynamics may influence team performance more than previously anticipated. In particular, recent studies in other academic disciplines suggest that social ties between team members may impact team dynamics but this has not been explored for medical teams. We aimed to explore intensive care staff’s perceptions about teamwork and performance in clinical emergencies focusing particularly on the teams’ social ties.

**Methods:**

Semi-structured interviews were conducted with a purposive sample of intensive care staff. We used a thematic analysis approach to data interpretation.

**Results:**

Thematic saturation was achieved after three group interviews and eight individual interviews. Findings demonstrated that social ties influenced teamwork by affecting the teams’ ability to co-construct knowledge, coordinate tasks, the need for hierarchy, the degree to which they relied on explicit or implicit communication, as well as their ability to promote adaptive behavior.

**Conclusions:**

Social ties may be an important factor to consider and acknowledge in the design of future team training, as well as for work planning and scheduling of team activities during clinical practice. More research is needed into the causal effect of social ties on team performance and outcome.

## Background

Time-sensitive emergency situations are common in intensive care units (ICUs). The intensive care team must be able to perform multiple interventions and make critical decisions in order to improve physical- and mental well-being for patients [1]. In the management of emergencies a multi-professional team approach is beneficial for efficiency as well as safety [1–3]. Nevertheless, coordination and communication can be challenging as the staff involved often have different professions, traditions and hierarchical status [4]. Emergency team members often favor a standardized approach using algorithms and protocols in managing emergency situations [5]. However, team members find it problematic to apply a structured approach when co-workers in ad hoc teams do not share the same knowledge, skills and experiences [4–6]. Although a variety of studies have demonstrated that team process behaviors, such as communication and coordination, contribute to effectiveness of clinical performance [7], there seem to be factors other than knowledge, skills, and attitude that influence team performance [8]. One of these factors could be the density of social ties within the team, which has been demonstrated to have positive influence on quality of treatment in emergency departments [9]. However, how social ties influence teamwork and performance has received limited attention in the context of clinical emergencies.

Social network theory provides one theoretical framework to help understand the influence of social ties within emergency team members. In social network theory, it is the *relationships* between individuals that are of interest rather than the specific attributes of individuals (e.g. knowledge and skills). Each individual in a network is considered as a ‘node’ in a complex interaction of ties between individuals [10]. These social ties may be expressive or instrumental. *Expressive ties* are those that relate to friendship and affective components whereas *instrumental ties* are those that arise in a professional setting [11]. In areas outside medical education, social network theory has been used to explain students’ academic performance based on strength of their interpersonal ties as well as to examine how employees interact with each other in the workplace [12]. When applied to health professions education, social network theory may enable a more collectivistic and holistic perspective to the analysis of teamwork instead of the traditional individualistic perspective focusing on individual team members’ competencies and performance.

The aim of this study was to explore staff’s perceptions of teamwork and performance in clinical emergencies with focus on the role of social ties within the team. To minimize the potential influence that context and team members’ knowledge and experience have on team performance [6], we chose the context of intensive care for our study as intensive care staff in general are experienced professionals familiar with managing clinical emergencies, such as resuscitation or difficult airway management.

The research question was: How do intensive care staff perceive the role of social ties in teamwork and performance?

## Methods

### Design

The study was explorative, using a constructivist, thematic analysis approach for data collection and analysis [13]. Data collection was performed in two iterations. We started with group interviews to gain a broader understanding of how task, context and social ties between team members affect their perceptions about team performance. Based on these initial findings, the interview guide was revised and we conducted individual in-depth interviews to gain a richer understanding of the role of social ties for teamwork in emergency settings. Consistent with a theoretical sampling approach, data collection continued until saturation of themes was achieved [14]. When saturation was met the full dataset was coded according to the final themes.

### Setting, time and participants

The interviewees were a purposive sample of intensive care nurses and intensive care physicians employed in two ICUs in Greater Copenhagen.

### The interview guide

Semi-structured interviews were performed. The first interview guide aimed to explore how factors related to people, task, and context affected aspects of teamwork and performance. The second interview guide, developed from the findings in the initial analysis, aimed at further exploring the role of social ties within the team. In our study the term social ties include both expressive ties and instrumental ties [11]. Both interview guides were developed in two steps. First, the project group discussed the content and questions and approved the versions used in the pilot tests. Second, the interview guides were pilot tested with intensive care nurses and physicians to avoid misinterpretation, misunderstanding and leading questions. The interview guides were adjusted according to the findings from these processes. Data from the pilot tests were not included in the final dataset.

### Data collection and data analysis

The three group interviews were conducted performing one interview every day 3 days in a row. The interviews were transcribed and the transcripts and the interviewers’ notes were analyzed for emerging patterns and themes related to the research question after each interview. Saturation was meet after three interviews.

A new semi-structured interview guide was developed, and the form was changed to individual interviews, as we wanted to learn more about the *expressive ties*, which we assumed the interviewees would speak more openly about during individual interviews. Eight individual interviews were conducted, two per day in 4 days over a two-week period. The interviews were transcribed and the transcripts were analyzed for emerging patterns and themes related to the research question after every two interviews. Saturation was met after eight interviews. Data was subsequently analyzed in multiple iterations until theme agreement was achieved. The full dataset was then coded according to the final overall themes using NVivo®.

## Results

### Interviews

Three group interviews and eight individual interviews were performed. The groups included mixed groups of nurses and physicians and the individual interviews included four nurses and four physicians. The full dataset included interviews with eight intensive care physicians and ten intensive care nurses, Table 1.

**Table 1:** Participant demographics, including interview method

	Interview (Group/individual)	Years of intensive care experience (Median (Range))	Years in department (Median (Range))
Nurses	6/4	10 (4–17)	10 (0.5–14)
Physicians	4/4	8 (2–18)	3 (0.2–15)
Total	10/8	9 (2–18)	7 (0.2–15)

### Themes

Four overall themes emerged, Table 2. One related to *social ties within the team*, the second to *team members’ knowledge and experience*, the third to *team coordination* and the fourth to *team communication*. In the following the interrelated themes are described separately but with the main focus on their complex link to social ties. As this link is our main focus, the theme, *social ties within the team*, is described first.

**Table 2:** Selected examples of interview citations. There are four overall themes

**Social ties within the team** The theme involves “having knowledge of someone” or “knowing and/or liking someone”.	
[P4]: It becomes an expectation of who it is I’m together with and I think that I will meter it out depending on whether it’s someone I know and have seen before or whether it’s someone I don’t know and haven’t seen before, and this goes for both physicians and nurses. [N5]: Agree, and I also think it’s right - that issue about turning to the physician you know—you know, in the situation. [P4]: Providing you feel comfortable with the person. [N4]: If we know each other, then I also know what’s expected of me.... it’s very nice to know that this and this physician expects that and that and that—also on some kind of personal level. So when we know each other... then things don’t need to be said. [P5] Well, it’s that we know each other well, the physicians and nurses; I think that always makes cooperation easier—you’ve tried something, maybe you’ve been in the same situations before. [N8] Of course it doesn’t matter if I like them privately, um, I’ve also been told off by one of the experienced physicians, one who’s been around for ages, who afterward came and apologized and I said: well, of course I know who you really are—you were just stressed out then. [N7]: I often think that you’re a little bit handicapped by it being so much word of mouth, you know, it’s as though there are some who are really quick to say: ”him, he doesn’t give a shit, watch out for him; or him, he’s ok”, you know it can, it can happen in some departments here, you know, quite quickly and I have always been very much against it and feel bad about it because you ”Borrow” it, you just can’t help listening, even if you don’t want to, because everybody should have a chance, if they’re interested and if they can manage.	
**Team member’s knowledge and experience** The theme covers aspects of knowledge and experience, including the use of standardized protocols and algorithms	
[P6] And then experience, of course, that’s the all-decisive thing, right? Experienced staff are not easier to cooperate with, but there are fewer things you need to say or discuss, right? N7] Well, you know, what’s it about, the optimal team, that would be some nurses who’ve been here just as long as I have and who I’ll know really well, because we don’t need to talk to each other so much. And then a physician, like, you know, some of these experienced consultants that we have, right? Who say: “ I want this and that”, and yes, then we dance like puppets around the physician, and then things just roll along. [P4]: It’s my impression that if you’re working in a setup where you know each other well and you’re familiar with the procedures you have to perform, then you get more practiced, it becomes less based on algorithms and more like you know each other and you suss out each other and you know what has to be done, um, and then I think that if you intuitively sense that is changing from what you’re used to… that’ll then be some sort of intuitive hunch… then you change your strategy and become more, um, how shall I put it, stringent or structured, I think.	
**Team coordination** The theme refers to the sequence and timing of activities, including task distribution, leadership and followership.	
[P6] Well, as a rule, I think it’s good in those situations that there’s this pre-defined role, so you don’t have to start by defining who’s doing what. That you automatically know that this, this is the area for my working skills, and then you get ready. People who can work and think independently. Um, and communicate, right? [P1]: You know, I don’t start by telling everybody who’s doing what, because I’m expecting everyone to have a purpose and that they know where we are heading. […] Actually, I don’t know if that would be of any help or if (reflective pause)… because I have this slight hunch that… but maybe it’s stupid not to do… you know, maybe it’s stupid to rely on the hunch… because it could be – that you are working with inexperienced staff. [N8] Whether they can take the lead, I think for nurses, well we know what’s needed but we’re not allowed to direct and if the physician is uncertain, then maybe the nurses also become uncertain but perhaps more irritated, because we know, and it’s not us who are to tell the physicians what they should do. [N7] We don’t have the responsibility, and it’s not that I couldn’t do it, but it’s also… um… I shouldn’t do it, I wouldn’t do it. [P4]: It can also be that it changes… you know, someone with a higher position and more experience comes along, you know, if it’s an intern or a resident who has the responsibility for the first time, then someone with a higher position comes in, and then most people, you know, provided the nurses are feeling comfortable with the person coming in, they will turn to that person. [N3]: When you don’t know each other, then the hierarchy becomes very prominent (illustrates this with arms raised and hands together pointing upwards), but here we have a flatter structure (lets arms fall), um, because we know each other—it’s not so hierarchical.	
**Team communication** The theme covers aspects of verbal and non-verbal communication as well as implicit and explicit ways of communicating.	
[P2]: Then, as always, comes the discussion about forms of communication. The way we talk to each other, and I think that it’s difficult to assess when you’re in the midst of it yourself. I can see that those times I’ve been in the trauma centre myself, there have been 16 people coming in, and that every single time, 5 of them—presumably—have never met before. There they have a very straightforward way of communicating also amongst themselves, but I think that when you’re there with people who you know really well, then it becomes much easier for me to talk in a more familiar way. You know, this does, of course, increase the risk that some things that are not being said are not done either—conversely, it also gives the nurse an opportunity to actually go and do something by themselves that I may have forgotten, or to come to me and ask about it. N8] The unspoken, when it really matters. Then, um, then I know them so well and they know me so well, um, and that’s also got something to do with the way I communicate—I don’t need to be so correct in how I communicate with them. If they know me well, then they also know whether I’m joking or not, right? [P4]: Whether it makes sense to drop the department’s way of doing things and do it the way it’s done in the trauma centre, and whether it can be a smooth transition or if you have to make a cut-off point and say that there are some things that we do here… there goes the bell and now we become different communicative people… I don’t know. I can only sense that, despite everything else, there is a smooth transition between when we are having coffee and when we are intubating together, but there’s no real decision that from now on we’re talking to one another in a different way. [P7] But I often find that if you’re in a place with a lot of people you don’t know, then the little things just aren’t being caught, and sometimes nobody does what’s been asked, and I know about closed loops and all that. But when you don’t know people, closed loops can be difficult. [N7] It’s more about what you feel comfortable with, because you know your colleagues, so you know that things are getting done. It’s another thing when they are new colleagues, then clear communication replaces the knowledge you have of your colleagues, well that’s what I think.	

### Social ties within the team

The interviewees emphasized the importance of knowing their co-workers. They described that communication with someone you know is easier, and that if you know your co-worker you tend to be more forgiving and helpful. If the *expressive ties* were strong, if they liked and trusted a co-worker, it was more likely that they would ask for advice or support, irrespective of profession, tradition and/or hierarchy. The interviewees mentioned that having personal knowledge of someone potentially had the same advantages as knowing someone professionally. Despite the importance of social ties during emergencies, the interviewees stressed that they were “professional enough” to put aside their social preferences. Strengthening of the social ties could be created by repeatedly working together or by simply “drinking coffee together” during a break. Furthermore, their individual perception of a co-worker could be modified by rumors and other co-workers’ opinions and their prior experiences from working together (reputation).

### Team member’s knowledge and experience

Team members’ individual knowledge and experience were highly valued by the interviewees and described as important for achieving effective team performance. However, there was a tendency toward nurses wanting a clear, fixed standard, whereas physicians appreciated more adaptable or “flexible” standards. Shared knowledge of specific department routines and protocols including algorithms was highly appreciated, even in situations where the team needed to deviate from the standards. In these situations, social ties helped the interviewees to trust, challenge and/or accept the decisions being made. The interviewees reported that *instrumental ties,* knowledge of co-workers’ prior experience and/or knowledge, made coordination and communication easier by enabling a more implicit communication and coordination of the teamwork.

### Team coordination

The interviewees emphasized the importance of having clearly defined roles. Knowing what to do next was described as reassuring, with potential to reduce cognitive efforts needed and to provide the possibility to pre-plan own tasks. However, the interviewees emphasized implicit coordination as being their preferred coordination style in most situations. Physicians stated that in case they *sensed* that the nurses were coordinating and performing the appropriate tasks, they would not interfere or explicitly approach the team with information or ask for specific tasks to be done. The interviewees felt that strong social ties within a team made the task distribution easier as *knowledge of co-workers* contributed with cues for intuitive assessment of skills, knowledge and experience.

The interviewees described the importance of hierarchies in relation to coordination. There were different types of hierarchy, one being flat versus a more top-down type, another being a formal versus an informal type—the formal being based on legal responsibility according to professional status, the informal based on experience and knowledge of department routines. The hierarchy was closely related to how well staff knew each other. When strong social ties were present the hierarchy flattened. The formal hierarchies came into play when staff for some reason sensed uncertainty, which could be related to lack of knowledge of the department’s normal routine or perceived lack of expertise or experience. In addition, the interviewees reported that those they would turn to or trust in a critical situation would be those they *knew* and *liked* the best, not necessarily those who were considered the best qualified.

### Team communication

Implicit communication was highly valued by staff. Non-verbal communication was appreciated, where staff were able to “sense” what their co-workers were doing or thinking and in which direction they were heading. In teamwork where staff knew their co-workers, explicit communication was perceived as less important. However, explicit communication was necessary when communicating with “unknown”/“new” or inexperienced co-workers or in situations where the team for some reason was losing the sense of direction. Staff reflected on which cues they used to decide when to change from implicit to explicit communication or vice versa, and it seemed to be highly individual, context-dependent and influenced by former experiences with similar cases or co-workers. Hierarchical status, formal or informal, as well as the atmosphere within the team had an influence on which communication style staff preferred. In emergency situations, where team members had strong social ties, the formal hierarchy was less important, which allowed the entire team to ask questions and come up with suggestions regarding the patient care.

## Discussion

The four themes and thus the combination of instrumental and expressive ties point in different directions: Social ties within the team emphasize the relational and social character of the group working with each other. Team member’s knowledge and experiences highlights the subject matter expertise of the team members, as perceived by their colleagues. Team coordination foregrounds how the team members used their knowledge about the situation at hand, about processes and guidelines, about what they know (or assume) their colleagues are able and willing to do, and about an overview of what was already done, what is currently done, and what will be done next. Team communication provides some insights into the many ways, how team members use a range of verbal and non-verbal cues to gauge their colleagues’ abilities and professional trustworthiness. In summary, the themes identified might shed some light onto those aspects beyond medical knowledge and skills that impact teamwork and possibly performance.

Staff valued when *they knew* that co-workers were familiar with the department’s routines and standards. This finding is in line with our earlier study, where former Advanced Life Support (ALS) course participants found it easier to work with co-workers, who also had attended an ALS course [5]. Emphasis on standardization of behavior through routines in coordination processes is common in medicine [14]. Nonetheless, the disadvantage of routines include that they may impede further learning, adaptation and flexibility [15]. This dilemma becomes overt in the interviews as physicians appreciate having adaptable standards, whereas nurses prefer fixed standards. Earlier studies on resuscitation teams have shown that teams with a more explicit communication and coordination style performed better than other teams [16]. However, our findings suggest that implicit and explicit team coordination modes have both advantages and disadvantages. It has been suggested that the coordination and communication modes should be adjusted according to the context [17] and this kind of adjustment is being practiced in some settings [18]. Nevertheless, staff in our study highly valued the implicit communication and coordination style.

Although our data do not explain why staff favored the implicit style there may be several explanations. One is that in order to work implicitly, team members need to have shared cognition of the situation as well as the skills and knowledge to perform the tasks required. Hence, implicit communication and coordination might be a proxy for knowledge and experience. The point here being that it is not the style of communication or coordination that staff value, but the underlying prerequisite for implicit coordination and communication.

The perception of hierarchy was closely related to social ties. In teams with strong ties the hierarchy flattened, whereas the need for a formal hierarchy was emphasized in teams with weaker ties. Nurses called for a clear leader who was willing and able to take responsibility. The wish for a flat hierarchy *with* a strong leader may seem contradictory. However, one explanation for this paradox could be that when staff members know who they are working with—including their profession, personality and their ability to make decisions—the need for an explicit hierarchy is counterbalanced by shared cognition and the extent of trust and ties in the team. Another explanation could be that there are two hierarchical levels: *the social level*:” we are all equal”; and *the task level*:” we do different things” that might influence how teams function differently depending on how strong the ties are within the team.

The interviewees in this study valued that social ties within the team would support their intuition, lower their cognitive efforts and help their decision-making. In clinical emergencies where time is a critical factor, professionals often trade decision accuracy for decision speed because of the resource intensiveness of rational decision-making [19]. The nature of this type of decision-making is therefore to make the best decision in a particular situation and to reassess and change direction if necessary. If an individual is socially related to a co-worker in advance, a benefit could be reducing the cognitive load [20]. However, a possible disadvantage could be being locked in previous assumptions and earlier patterns of interaction [21]. When staff do not know their co-workers, intuitive assumptions must be made of who to trust [22] combined with a *sense* of who possesses which knowledge and skills [4]. Staff reported that those they would turn to or trust in a critical situation would be those whom they had the strongest expressive ties with - *those they knew and liked the best* - and not necessarily those they considered the best qualified. This finding is in line with an earlier study where nurses claimed to involve people who they were most comfortable with rather than those who would be the best to solve the problem [23].

### Limitations of the study

The study has some limitations. There is an issue related to the applicability in other contexts. Intensive care staff is likely to have prior experience of working together, which might be an important contrast to other emergency settings where multi-professional teams join from different departments ad hoc. Accordingly, the findings might not transfer to other multi-professional team settings. Yet, the fact that social ties, *even* in the ICU setting, were described as being an important factor influencing teamwork could indicate an even stronger influence in other settings. In this study, we did not explore the relation between social ties and teamwork performance. A recent study of college students showed that a team’s performance was strongly correlated with the teams’ social ties and that the strongest ties explained more of the variance in performance than other factors such as team members’ personalities and competencies [12]. Future studies are needed to explore to what extent strong social ties between team members are associated with better team performance and better patient outcomes within the context of clinical emergencies.

The implication of social ties for instructional strategies during teamwork could be of importance in the design and planning of future team training activities.

## Conclusion

Social ties, instrumental as well as expressive, influence teamwork in managing clinical emergencies by affecting the teams’ ability to co-construct knowledge, their task coordination, the need for hierarchy, the degree to which they relied on explicit or implicit communication forms, as well as their ability to promote adaptive behavior.

Social ties may be an important factor to consider in the design of future team training, as well as for work planning and scheduling of team activities during clinical practice.

## Additional file

**Additional file 1.** Interview guide 1 and 2.

## Data Availability

Transcripts of interviews can be sent upon request by contacting the first author.

## Abbreviations

ALS: Advanced Life Support
ICU: Intensive Care Unit
