# Supplementary material for: Social ties influence teamwork when managing clinical emergencies
Source: BMC Med Educ. 2020 Mar 4;20:63. doi: 10.1186/s12909-020-1953-8 (PMC7057460; doi:10.1186/s12909-020-1953-8)
Supplement: Supplementary file 1 — Additional file 1. Interview guide 1 and 2. [file 12909_2020_1953_MOESM1_ESM.docx]

**Interviewguide 1**

My name is MBR and I am a physician and a PhD student. I am PD and I am a psychologist and MBRs supervisor/wingman. We currently work on a study aiming to explore team member’s actions in time pressured situations. We expect that the interview will take approximate 30 minutes. We will record the interview – which I hope you’ll all approve.

1. My first question concerns demographics, and I would like to know your professions, intensive care experience (in years) and how long you have been employed in this particular department.

2. Then I would like you to consider a clinical situation you have encountered, where you have been under time-pressure. Preferably a situation that went well.

3. Then I would like you to consider a clinical situation you have encountered, where you have been under time-pressure. Preferably a situation that went not so well.

5. Is there anything you would like to add?

Thank you so much for your time and for participating.

**Interviewguide 2**

My name is MBR and I am a physician and currently I work on a study aiming to explore team member’s actions in time pressured situations. I expect that the interview will take approximate 10-15 minutes and I will record it – which I hope you will approve.

1. My first question concerns demographics, and I would like to know your profession, intensive care experience (in years) and how long you have been employed in this particular department.

2. Then I would like you to consider a clinical situation you have encountered, where you have been under time-pressure. Preferably a situation that went well.

3. Then I would like you to consider a clinical situation you have encountered, where you have been under time-pressure. Preferably a situation that went not so well.

4. Could you describe your ”golden” team? Who (not specific persons) is on it, and why? Which competences do they have?

5. Is there anything you would like to add?

Thank you so much for your time and for participating.
